# Monascus purpureus induced apoptosis on gastric cancer cell by scavenging mitochondrial reactive oxygen species

**DOI:** 10.3164/jcbn.17-27

**Published:** 2017-09-26

**Authors:** Hiromi Kurokawa, Hiromu Ito, Hirofumi Matsui

**Affiliations:** 1 Faculty of Medicine, University of Tsukuba, 1-1-1 Tennodai, Tsukuba 305-8575, Japan; 2 Graduate School of Medical and Dental Sciences, Kagoshima University, 8-35-1 Sakuragaoka, Kagoshima 890-8544, Japan; 3 Kyoto Prefectural University of Medicine, Kamigyo-ku, Kyoto 602-8566, Japan

**Keywords:** Monascus purpureus, mitochondrial reactive oxygen species, apoptosis, acid ceramida

## Abstract

Monascus purpureus is a red dye derived from yeast rice and has been used as color additives for food in East Asia. Monascus purpureus consists of several bioactive components. Some of these components work as a radical scavenger, thus monascus purpureus would also eliminate reactive oxygen species. Cancer cells maintain the high level of reactive oxygen species than normal cell and are death by imbalance in pro-oxidant/antioxidant homeostasis. In this study, we investigated whether monascus purpureus induced cancer specific cell death by scavenging reactive oxygen species. Compared to normal cell, monascus purpureus had cancer specific cytotoxicity. Monascus purpureus and lovastatin, its component, scavenged free radicals caused by a xanthine/xanthine oxidase system, thus Monascus purpureus is likely to scavenge reactive oxygen species by a synergistic effect between lovastatin and other components. Monascus purpureus also decreased reactive oxygen species derived from mitochondria in cancer cells, and cellular apoptosis was induced via activation of caspase-9. Induction of apoptosis by reduction of reactive oxygen species generation decreased acid ceramidase, and this mechanism could be involved with increasing ceramide accumulation in cells.

## Introduction

Monascus purpureus (MP), a red dye derived from yeast rice, is known as *beni-koji* and has traditionally been used to make food more colorful in East Asia.^(1)^ Some bioactive components from MP have been identified and characterized such as monacolin K, γ-amino butyric acid (GABA), dimerumic acid and ankaflavin. Monacolin K^(2,3)^ has been demonstrated as specific inhibitors of both 3-hydroxy-3-methylglutaryl-coenzyme A (HMG-CoA) reductase, a rate-limiting step in cholesterol synthesis and has the same chemical structure as lovastatin which is known as a hypolipidemic agent. GABA possesses several physiological functions such as hypotension, antidepressant effect and neurotransmition.^(4–7)^ Dimerumic acid has radical scavenging ability and can scavenge radical dose-dependently.^(8)^ Ankaflavin is also radical scavenger, and has anti-inflammatory and anti-tumor effect.^(8)^

One of the important roles of reactive oxygen species (ROS) is regulating physiological signaling pathways to proliferate and differentiate.^(8)^ Oncogenes, mitochondrial mutations, hypoxia, or tumor suppressor loss have been reported to induce production of mitochondrial ROS (mitROS) which involve tumorigenic signaling.^(9)^ We have reported that the relation between mitROS and characteristics of cancer using a rat cancerous gastric mucosa cell-line (RGK1) and manganese superoxide dismutase (MnSOD)-overexpressed RGK cells, RGK-MnSOD. Migration and invasion in RGK1 cells increased as compared to RGK-MnSOD, thus we concluded that mitROS accelerate cancer cell invasion.^(10)^ Maintenance of high ROS concentration is essential for cancer cells to proliferate, however an excess of ROS induces cytotoxicity. To avoid this problem, ROS scavenger such as SODs and catalase in cancer cells works to prevent cell cytotoxicity. On the other hands, low ROS concentration lacks the ROS signaling to maintain growth and induce cytostasis. Balance between ROS level and antioxidant activity is very important in cancer cellular homeostasis, thus cancer therapies, which is involved in increase or decrease of intracellular ROS level, are studied.^(11–13)^

Many researchers have been investigating on cancer therapy using antioxidants. From the point of view of the low risk for side-effects, natural antioxidants from food materials are more useful.^(14)^ Yao *et al.*^(15)^ reported that cell growth in cancer cells was inhibited by curcumin in a dose-dependent manner. Ben *et al.*^(16)^ reported that rutin inhibited proliferation and decreased adhesion and migration in cells. Kim *et al.*^(17)^ reported that ascorbic acid inhibited p53-induced senescence by preventing ROS generation and p38 MAPK activity to inhibit cancer cellular proliferation.

It is reported that MP components induce cancer cell death. However, the detailed mechanism remains unclear. MP may work as the ROS scavenger because its components are antioxidants. Moreover, the less-harmfulness of MP has already been confirmed because it has been casually used as a color additive for food. In this study, we investigated that whether MP worked as a ROS scavenger and involved cancer specific cytotoxicity.

## Materials and Methods

### Materials

Monasucus pigment, hypoxantine and malic acid were obtained from Wako Pure Chem. Ind., Ltd. in Japan. Xanthine oxidase (Roche, Basel, Switzerland), 2-[5,5-dimethyl-2-oxo-2λ5-(1,3,2)dioxaphosphinan-2-yl]-2-methyl-3,4-dihydro-2H-pyrrole 1-oxide (CYPMPO; Radical Research Inc., Tokyo, Japan), 5,5-dimethyl-1-pyrroline *N*-oxide (DMPO; Labotec, Tokyo, Japan), β-nicotinamide adenine dinucleotide (NADH), d-glutamic acid, succinic acid and Trizma^®^ base, Triton X-100 and Tween^®^ 20 (Sigma-Aldrich Japan K.K., Tokyo, Japan), Cell Counting Kit-8, MitoSOX and Hoechst 33258 (Dojindo, Japan), deoxycholic acid and hydrochloric acid (Wako Pure Chem. Ind., Ltd., Osaka, Japan), NuPAGE^®^ Novex^®^ 12% Bis-Tris gels (Life Technologies Japan Ltd., Tokyo, Japan), PVDF Blocking Reagent for Can Get Signal^®^, Can Get Signal^®^ Immunoreaction Enhancer Solution 1 and Can Get Signal^®^ Immunoreaction Enhancer Solution 2 (TOYOBO Co. Ltd., Osaka, Japan), caspase-9 antibody and horseradish peroxidase (HRP) linked anti-rabbit IgG antibody (Cell Signaling Technology Japan, K.K., Tokyo, Japan), ASAH1 antibody (Abcam plc., Cambridge, UK), Lumina forte western HRP substrate (Millipore Co., Billerica, MA), lovastatin (Tokyo chemical industry CO., LTD., Tokyo, Japan), MitoSOX (Life Technologies Inc., Gaithersburg, MD) were purchased and used without further purification or modification.

### Cell culture

RGK1, RGK-MnSOD and RGK vector cells were cultured in DMEM/F12 without l-glutamine (Sigma-Aldrich Japan K.K.). Rat normal gastric mucosal cell line RGM1 cells were cultured in DMEM/F12 with l-glutamine (Life Technologies Japan Ltd.), respectively. These culture mediums contained 10% heat-inactivated fetal bovine serum (Biowest, Kansas City, MO) and 1% penicillin/streptomycin (Life Technologies). Cells were cultured under 5% CO_2_ at 37°C.

### Cell viability test by WST assay

Cell viability was examined using the Cell Counting Kit-8 according to manufacturer’s protocol.^(18)^ RGK1, RGK-MnSOD, RGK vector and RGM1 cells were cultured on 96-well plates at 5 × 10^3^ cells/well and incubated overnight. The supernatant was then aspirated and the medium was replaced. To determine the cytotoxicity of intracellular MP, these media contained 0, 50, 100, 150, 200, 250 and 300 µg/ml of MP respectively. Cells were incubated at 37°C for 20 h. After incubation, cells were rinsed twice with phosphate-buffered saline (PBS), then incubated with 10% Cell Counting Kit-8. The absorbance at 450 nm was measured by a DTX880 multi-mode microplate reader (Beckman Coulter, Brea, CA).

### Electron spin resonance spectroscopy

Reactivity of MP against ROS was measured by ESR. The scavenging activities of MP and lovastatin in solution were estimated using CYPMPO. Superoxide anion radicals were generated from a xanthine/xanthine oxidase (X/XO) system. This reaction mixture contained phosphate buffer with 20 mM hypoxanthine, 20 units/ml xanthine oxidase and MP or lovastatin. The scavenging activity of MP in cancer cells was also estimated using DMPO. RGK1 RGK-MnSOD and RGK vector cells were incubated until confluence, then cells were treated with 0, 100 or 200 µg/ml MP for 24 h. After incubation, cells were suspended with a respiratory solution (5 mM succinate, 5 mM glutamate, 5 mM malate, and 5 mM NADH) containing a spin-trapping agent [5.9% (v/v) DMPO]. The solution was immediately transferred to a quartz flat cell (60 × 6 × 0.3 mm; RDC-60, Radical Research). The electron spin resonance (ESR) spectra were recorded using a JEOL-TE X-band spectrometer (JEOL, Tokyo, Japan). ESR spectra of MP and lovastatin in solution were obtained under the following conditions: 20 mW incident microwave power, 9.2 GHz frequency, 0.2 mT modulation width, 7.5 mT sweep width, 0.1 s time contrast and 335.5 mT center field. ESR spectra of RGK1 cells were obtained under the following conditions: 10 mW incident microwave power, 9.4 GHz frequency, 0.1 mT modulation width, 7.5 mT sweep width, 0.1 s time contrast and 335.5 mT center field.

### Measurement of mitochondrial ROS

MitROS were detected using a fluorescence indicator, MitoSOX. RGK1 cells were treated with 200 µg/ml MP or without for 20 h. After the treatments, cells were incubated for 15 min in 5 µM MitoSOX diluted with Hanks’ balanced salt solution (HBSS). After incubation, the cells were washed three times with a HBSS. Fluorescence intensity of MitoSOX was measured by a Varioskan micro plate reader (Thermo Fisher Scientific K.K., Kanagawa, Japan). The measurement wavelength of excitation and emission were 510 nm and 580 nm, respectively.

### Detection of apoptotic cells by Hoechst 33258 staining

The apoptosis of RGK1 cells was detected using the Hoechst 33258. RGK1 cells were incubated with or without 200 µg/ml MP for 20 h. Cells were detached with trypsin and incubated with Hoechst 33258 for 30 min. After treatment, cells were put on the cover glass. Apoptosis, with condensed and fragmented nuclei, was analyzed using a fluorescence microscope (ECLIPSE Ti2, Nikon Instruments Inc., Tokyo, Japan) under 40× objective lens (S Plan Apo 40 × 0.60, Nikon).

### Western blotting analysis

Cells were culutured overnight at 37°C. Fresh medium containing 200 µg/ml MP was then added and incubated for 24 h. After incubation, whole cell lysates were prepared by rinsing the cells three times with PBS, adding NuPAGE LDS Sample buffer on ice, then heating at 70°C for 10 min. For SDS-polyaclylamide gel electrophoresis, the cell lysates were added into wells of NuPAGE^®^ Novex^®^ 12% Bis-Tris gels. The gel was electrophoresed at 100 V for 70 min and proteins were transferred onto a PVDF membrane by electrophoresis at 1.2 mA/cm^2^ for 60 min. The membrane was blocked for 60 min with PVDF blocking reagent for Can Get Signal^®^ and probed with primary and secondary antibodies. Anti-rabbit caspase-9 or ASAH1 antibody (1:1,000) was added to the Can Get Signal^®^ Immunoreaction Enhancer Solution 1. The solution was exposed to the membrane overnight. After the primary antibody solution was aspirated, the membrane was washed three times by 1× Tris-buffered saline containing Tween 20. The secondary HRP-linked anti-rabbit IgG antibody was added to the Can Get Signal^®^ Immunoreaction Enhancer Solution 2. The solution was exposed to the membrane for 60 min. Chemiluminescence was used to develop the membrane. Images of the blots were captured by an ImageQuant LAS4000 (GE Health Care Japan, Tokyo, Japan). β-Actin was detected as the control for protein loading.

### Statistical analysis

Data are expressed as means ± SD and were assessed by analysis of variance. Individual groups were compared by Tukey’s post-hoc test or student *t* test with *p*<0.05 considered statistically significant.

## Results

### Cytotoxicity of MP in RGK1 and RGM1 cells

**Fig. 1:**
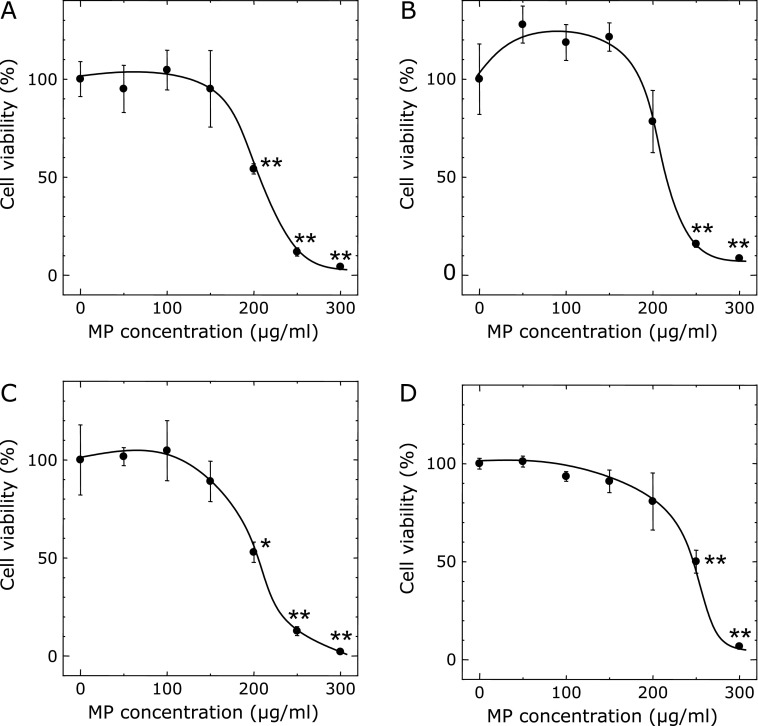
Cytotoxicity of MP. RGK1 (A), RGM1 (B), RGK vector (C) and RGK-MnSOD (D) cells were exposed to culture medium containing several concentrations of MP for 20 h, then WST assay was performed. Data are expressed as means ± SD (*n* = 4). *p<0.05, **p<0.01.

The cytotoxicity of MP in RGK1 and RGM1 cells were evaluated by the WST assay. Cell viabilities after treatment with 200 µg/ml in RGK1 and RGK vector were significantly decreased compared to control, while it in RGM1 and RGK-MnSOD were 78% and 81%, respectively. Cell viabilities after treatment with 250 and 300 µg/ml in all group cells were significantly decreased compared to control (Fig. 1).

### MP plays a role in scavenging of ROS

**Fig. 2:**
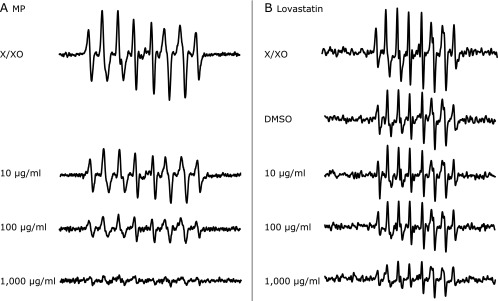
ESR spectra of ROS spin adducts generated by 20 mM hypoxanthine/20 U/ml xanthine oxidase. Each solution contains (A) MP or (B) lovastatin.

**Fig. 3:**
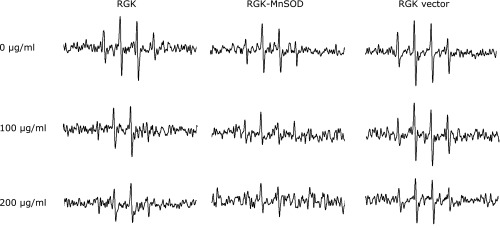
ESR spectra from RGK1, RGK-MnSOD and RGK vector cells after MP exposure. The intensity of ESR signals decreased when cells were treated for 24 h with or without MP. DMPO was used as the spin-trapping agent.

**Fig. 4:**
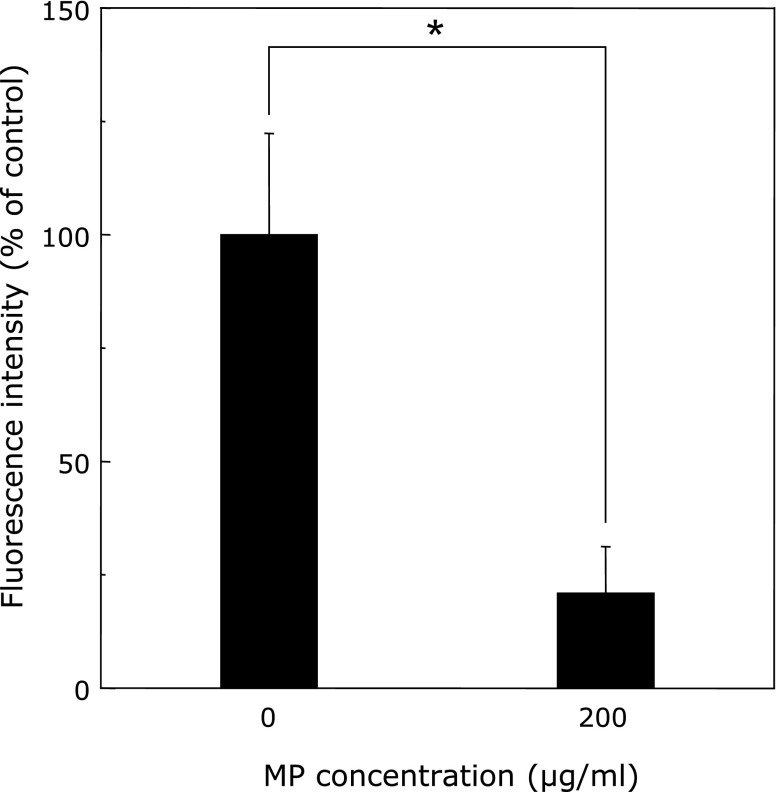
Fluorescence intensity of MitoSOX. RGK1 was treated for 20 h with 200 µg/ml MP or without MP. Ex. 510 nm and Em. 580 nm. Data are expressed as means ± SD (*n* = 4). *p<0.01.

We demonstrated the effect of MP as a ROS scavenger using ESR spectroscopy. Xanthine/Xanthine oxidase (X/XO)-induced ROS were scavenged by MP under cell-free condition in a dose-dependent manner (Fig. 2). Lovastatin, one of the MP components, also scavenged ROS in a dose-dependent manner (Fig. 2). We also examined whether MP could scavenge ROS in RGK1 cells. RGK1 and RGK vector cells treated without MP produced high level of ROS, while cells treated with MP suppressed production of ROS in a dose dependent manner (Fig. 3). ROS production in RGK-MnSOD was lower than that of RGK1 and RGK vector without MP treatment. However, compared to ESR signal without MP, suppression of ROS production by MP in RGK-MnSOD was lower than that of RGK1 and RGK vector. Moreover, it was confirmed that MP decreased mitROS specifically by use of MitoSOX, which is a mitochondrial superoxide indicator (Fig. 4).

### MP induces apoptosis in RGK1 cells

**Fig. 5:**
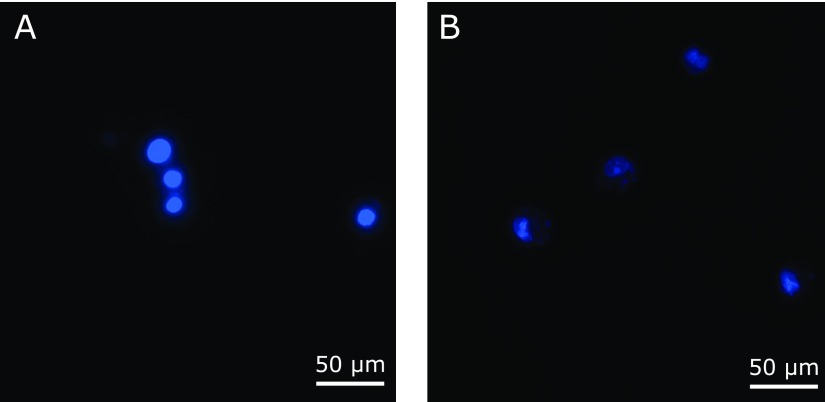
Hoechst 33258 staining of RGK1 cells. The cells undergoing apoptosis demonstrated apoptotic chromatin changes: blebbing, fragmentation and condensation under a fluorescence microscope. Cells were stained (A) without MP or (B) with 200 µg/ml MP for 20 h. Data were obtained in three independent experiments. The scale bar indicates a distance of 50 µm.

RGK1 cells were treated with 200 µg/ml MP for 20 h, thereafter cell apoptosis was confirmed by Hoechst 33258 staining assay. Morphological changes indicating cell apoptosis such as condensation of both chromatin and nuclear fragmentations were found only in MP treated cells. The result showed that MP treatment induced cancer cellular apoptosis (Fig. 5).

### Activation of caspase-9 and inhibition of acid ceramidase expression by MP

**Fig. 6:**
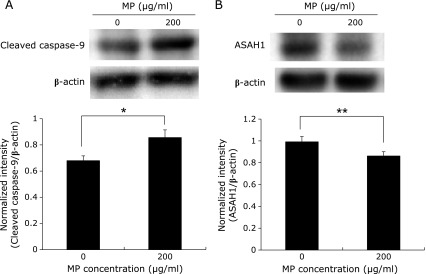
Western blotting analyses. (A) Cleaved caspase-9 protein expression. (B) ASAH1 protein expression. Data are reported as the means ± SD (*n* = 4). *p<0.05, **p<0.01.

RGK1 cells were treated with 200 µg/ml MP for 20 h and further analyzed for the cleaved caspase-9 and acid ceramidase by western blotting (Fig. 6). With regard to caspase-9 activation, treatment of MP exhibited significant increases of cleaved caspase-9. On the other hand, the expression of acid ceramidase was significantly decreased by the MP treatment.

## Discussion

In this study, we demonstrated for the first time that MP can scavenge mitROS to induce a cancer specific cell death. As mentioned above, maintenance of intracellular ROS concentration is important for cancer cells because an inadequate ROS concentration, more or less, is lethal. MP extractions have been reported to show a radical scavenging ability. Taken together, MP can be a reagent to induce cancer cellular injury to destroy a suitable ROS concentration by its radical scavenging ability. We investigated a cancer specific cytotoxicity by MP. After treatment with 200 µg/ml, cell viability in RGK1 cells was significantly decreased compared to control, while cell viability in RGM1 and RGK-MnSOD cells did not. Su *et al.*^(19)^ reported that ankaflavin, one of the MP component, was found to be toxic to human cancer cell lines Hep G2 and A549, while it posed no significant toxicity to normal MRC-5 and WI-38 cells at the same concentration. From our results, MP also showed higher cytotoxicity in cancer cells than in normal cells. Moreover, it was suggested that cancer cell death by MP treatment was effected by mitROS.

We considered cancer specific cell death by MP was induced through failure of ROS balance because MP extractions work as a ROS scavenger. Among ROS, superoxide anion (O_2_^•−^) is the precursor of almost ROS and is formed by the single electron reduction of oxygen. O_2_^•−^ induces activation of glycolysis and hypoxia-inducible factor 1 and can produce malignant transformation of cancer.^(20)^ Therefore, we investigated the relations between MP and O_2_^•−^ which can be generated in a simple reaction with hypoxanthine and xanthine oxidase in a cell free system. The generated O_2_^•−^ was measured by ESR with a spin-trap agent, CYPMPO.^(21)^ The generation of O_2_^•−^ from hypoxanthine/xanthine oxidase was reduced by MP in a dose-dependent manner (Fig. 2).

MP has been reported to be consisted several molds, parts of which show radical scavenging abilities. Aniya *et al.*^(22)^ reported that among 31 species of the molds monascus, the scavenging action was observed in 13 molds. Yang *et al.*^(23)^ reported that ankaflavin has protective effects against ischemia-reperfusion injury through anti-oxidant, anti-inflammatory, and anti-apoptotic mechanisms in mouse. Tseng *et al.*^(24)^ reported that MP NTU 568-fermented products were tolerative against oxidative stress. In this study, we also investigated about antioxidant effect of lovastatin that has the same chemical structure as monacolin K.^(25)^ Lovastatin works not only hypolipidemic agent but also antioxidant. Lin *et al.*^(26)^ and Song *et al.*^(27)^ reported that monacolin K decreased intracellular ROS production. Indeed, lovastatin reduced ROS that were produced by hypoxanthine/xanthine oxidase (Fig. 2). ROS scavenging capacity of MP was higher than that in lovastatin. We suggested that the antioxidative effect in MP was enhanced by a synergistic effect between lovastatin and other molds.

Since MP can work as a ROS scavenger which decreases intracellular ROS concentration to derive an imbalance environment, we considered that MP induce the cancer specific cytotoxicity. To prove this hypothesis, we examined the influence of MP on ROS level in RGK1 cells. ESR investigation confirmed that intracellular ROS level was decreased by MP in a dose-dependent manner (Fig. 3). ESR signal in RGK1 with 200 µg/ml MP was potently decreased compared to without MP treatment. However, ESR signal in RGK-MnSOD with 200 µg/ml MP was slightly decreased. Moreover, mitROS significantly decreased after the treatment with 200 µg/ml MP (Fig. 4). The intracellular ROS level can affect the phosphoinositide 3-kinase (PI3K) pathway, which induces to initiate cell proliferation, promote survival, and increase cellular mobility in cancer cells.^(28)^ ROS inactivate phosphatase and tensin homologue (PTEN), known as a PI3K negative regulator.^(29)^ Akt is activated by PI3K and phosphorylate several protein to regulate cell proliferation and survival.^(30)^ Pelicano *et al.*^(31)^ reported that mitROS inhibited PTEN and activated Akt specifically. Cell apoptosis via caspase-9 was induced by Akt inactivation, thus mitROS decrease would initiate apoptosis. Indeed, curcumin, one of a ROS scavenger, activates caspase-9 via ROS inhibition.^(32)^ We also observed chromosome condensation by Hoechst 33342 assay and increasing cleaved caspase-9 by western blot in cancer cells after treated with 200 µg/ml MP (Fig. 5 and 6). From these results, we concluded that MP induced cancer cell apoptosis by reducing mitROS.

Ceramide is formed from hydrolysis of membrane sphingomyelin to regulate cell growth, proliferation and apoptosis signal.^(33)^ Ceramide changes into protein phosphatases 2A (PP2A) to inactivate anti-apoptotic proteins such as BCL2 and AKT, leading to mitochondrial apoptosis by dephosphorylation.^(34)^ Lysosomes and endoplasmic reticulum are also involved in apoptosis.^(35)^ Ceramide is phosphorylated by a ceramide kinase such as acid ceramidase, thereafter sphingosine is produced by ceramidase action and phosphorylated to generate sphingosine-1-phosphate (S1P), is important for cell survival. Thus, decreasing acid ceramidase and increasing S1P aid cell survival and proliferation. Indeed, acid ceramidase is overexpressed in several cancer cells.^(36,37)^ Thangavel *et al.*^(32)^ reported that expression level of acid ceramidase decrease by ROS scavenger. In this study we also confirmed that the expression on acid ceramidase decreased after the treatment with MP: we used ASAH1, which is a gene to encode the acid ceramidase. Camacho *et al.*^(38)^ reported that cancer cells with ASAH1 inhibitors caused a dose-dependent accumulation of ceramides and inhibited their growth. Thus we proposed that the decrease of acid ceramidase expression by MP induced ceramide accumulation to involve cancer cellular apoptosis.

In conclusion, compared to normal cell, MP has cancer specific cytotoxicity. MP works as a ROS scavenger by a synergistic effect between lovastatin and other components. Since MP decreased mitROS, cell apoptosis was induced via activation of caspase-9. Cell apoptosis was induced by decreased acid ceramidase via ROS reducing and this mechanism involved by increasing ceramide accumulation.
